# Frequency of complete remission after standard 3+7 induction therapy in patients with acute myeloid leukemia

**DOI:** 10.12669/pjms.38.5.5249

**Published:** 2022

**Authors:** Iram Shireen, Sumra Komal, Abida Mateen Ansari, Lubna Meraj

**Affiliations:** 1 Iram Shireen, MBBS, FCPS. Post-Graduate Trainee, Medical Oncology, Jinnah Hospital, Lahore, Pakistan; 2 Sumra Komal, Pharm-D, M.Phil. Researcher, Department of Pharmacology, School of Basic Medical Sciences, Zhengzhou University, Zhengzhou, 450001, China; 3 Abida Mateen Ansari, MBBS, FCPS. Assistant Professor, Department of Medicine, Al-Nafees Medical College and Hospital, Islamabad, Pakistan; 4 Lubna Meraj, MBBS, FCPS. Associate Professor, Department of Medicine, District Headquarter Hospital Rawalpindi, Rawalpindi, Pakistan

**Keywords:** Acute Myeloid Leukemia, Complete Remission, Induction Chemotherapy, LDH, TLC

## Abstract

**Objectives:**

To find the frequency of complete remission rate after standard 3+7 induction therapy in patients with Acute Myeloid Leukemia (AML) among different clinicopathological groups.

**Methods:**

Non-probability purposive sampling technique was used to collect data from July 2016 to Jan 2017, conducted at Department of Oncology, Jinnah Hospital Lahore. Sample size of 50 cases was calculated with 95% confidence level, 14% margin of error and taking expected percentage of complete remission (CR) 57% in AML patients after 3+7 induction therapy.

**Results:**

Out of 46 patients, majority had AML-M2. AML- Not Otherwise Specified (NOS) disease. Most common pathological presentation was Total Leucocyte Count (TLC) of <50,000/mm^3^ (60%) as compared to patients with hyperleukocytosis i.e., TLC >50,000/mm^3^ (40%). Twenty-two percent had low Lactate Dehydrogenase (LDH) level involvement and 78% had high LDH level involvement. As compared to target of 57 %, complete response was observed in 54% patients, (p<0.02) with better results in younger age group, male patients with low LDH and TLC level.

**Conclusion:**

It was concluded that 3+7 induction chemotherapy has 54% CR rates in patients with AML. Whereas, in AML-M5, AML-M6, AML-NOS patients especially, with high LDH and TLC and patients with advanced age, CR rate is low and needs more aggressive treatment.

## INTRODUCTION

Acute myeloid leukemia (AML) is the most common type of leukemia with the worst prognosis. It is associated with the presence of >20% of blasts in peripheral film or bone marrow biopsy.^1^ Acute myeloid leukemia in the adult age group exhibits the worst prognosis with a 5-year survival rate of 15-20% with no difference in prognosis.^2^ Curative treatment for AML is based on fact to decrease leukemic burden by inducing complete remission (CR) (presence of <5% blasts in the bone marrow) with intensive cytotoxic chemotherapy to prevent relapse.^3^

In AML, standard chemotherapy regimen includes induction with cytarabine (100-200 mg/m^2^ for seven days) and daunorubicin (60-100 mg/m^2^ for three days), followed by consolidation chemotherapy with post-remission therapies such as hematopoietic stem cell transplantation (HSCT) to obtain long-term outcome. In resistant or recurrent cases, graft versus leukemia-based allogenic transplantation harness the effect produced by standard chemotherapy regimens.^4^ The first 4-week period of induction and post induction time is indicator of the prognosis. Similarly, mortality during this period is the indicative of treatment related mortality.^5^ Prediction of achievement of CR rate and mortality after first induction is done by different risk factors.^6^ Clinical, pathological and genetic factors play an important role in the prediction of response towards treatment. Poor risk patients are directed after CR, towards bone marrow transplant or clinical trials.

Patients with poor prognostic histological factors include the French-American-British (FAB) classification system, AML-M4, M5, and dysplasia. Pathological factors or indicators of high tumor burden and poor prognosis include high TLC count and LDH level.^7^,^8^ At present, there is no such study in Pakistan to assess clinical response after standard induction. Therefore, the current study focused on the induction therapy response rates taking into account different factors such as age, gender, LDH level, hyperleukocytosis (TLC count >50,000/mm^3^), Eastern Cooperative Oncology Group (ECOG) status, and FAB class.

## METHODS

Sample size consisted of fifty (n=50) patients diagnosed with AML with curative intent admitted in Medical Oncology department of Jinnah hospital Lahore from July 2016 to January 2017. Four patients were not able to complete the 3+7 regimen due to life-threatening febrile neutropenia resulting in infections, hence were dropped from data. Informed consent was signed by the patients before the commencement of treatment. Patients were assessed (before starting standard induction chemotherapy) with history and general physical examination. It had been complemented with investigations like complete blood counts (CBC), liver function test (LFT), renal function tests (RFT), beta 2 micro globulin (B2M) levels, LDH, serum uric acid, abdominal ultrasound, chest x-ray, echocardiography (to check pre-anthracycline cardiac status) and cytogenetics. TLC >50,000 /mm^3^ had been considered as hyperleukocytosis.

### Inclusion and Exclusion Criteria:

Inclusion criteria include the patients recently diagnosed with AML 0 - 7 (Excluding AML -M3) of both genders with age between 16 and 60 years and exclusion criteria those with secondary AML (drug-induced / myelodysplasia), CNS involvement, or soft tissue disease (chloroma), poor performance status and severe infection (high-grade fever >100F), tachycardia (>100 beats per minute), hypotension (Systolic Blood Pressure <60 mm Hg). Further, there were four treatment-related mortalities and therefore excluded from the trial.

### Response Assessment:

Response in terms of complete response (CR) and no complete response (CR) was evaluated as per chosen criteria after seven days of last chemotherapy with bone marrow, CBC, and clinical examination (on day 14). After three weeks of completion of induction chemotherapy the response was documented as either CR (<5% blasts in bone marrow examination) or no complete response (no CR) more than 5% blasts as per bone marrow analysis of the blasts percentage as per chosen criteria.

### Statistical Analysis:

Statistical analysis was done by using SPSS 21.0 (SPSS Inc., Chicago, IL, United States). Quantitative variables like age are presented as mean and standard deviation. Qualitative variables like gender and disease response (complete, no complete response) are presented as frequency and percentage. Data stratified by age, gender, FAB class, TLC & LDH level to address effect modifiers. Post-stratification chi-square test was applied to test the statistical significance of observed variation in responses in different groups with a P-value ≤0.05 as significant.

## RESULTS

Out of 46 patients, 12 (26%) had AML-M2, and 10 (21%) patients had AML-NOS subtype, and 9 (19%) had AML-M4 at baseline. The most common presentation was the low TLC count i.e., a total of 28 patients (60%) had low TLC count and low LDH involvement in 36 (78%) patients. The most common complaint was fever in 17 (35%) patients (Table-I).

**Table I:** Distribution of demographic characteristics of study population (N=46).

Demographic Characteristics	No of Patients N (%)
**Gender**	
Male	34 (74%)
Female	12 (26%)
**Age**	
Young (< 40 years)	30 (65%)
Old (> 40 years)	16 (35%)
**Clinical Presentation**	
Fever	17 (35%)
Fatigue	8 (18%)
Bleeding	12 (28%)
Leukocytosis	5 (10%)
Extra medullary involvement	4 (9%)
**LDH**	
Low LDH (<400 U/L)	36 (78%)
High LDH (>400 U/L)	10 (22%)
**TLC**	
High TLC (> 50,000 mm^3^)	18 (40%)
Low TLC (<50,000 mm^3^)	28 (60%)
**ECOG Performance Status**	
Good	32 (69%)
Poor	14 (31%)
**FAB Class**	
M1	6 (12%)
M2	12 (26%)
M4	9 (19%)
M5	3 (6%)
M6	4 (8%)
M7	2 (4%)
NOS	10 (21%)

Patient’s response to induction chemotherapy was assessed after 21 days of completion of the cycle of chemotherapy and a complete response was observed in 25 (54%) patients, compared to the target complete response rate of 57%. No CR was observed in 21 (46%) patients, this result was statistically significant with P<0.02.

Study population was stratified according to FAB class, age, gender, LDH and TLC and performance status. Response rates were also assessed separately for different groups. The overall responses and percentages for different groups with their respective P-values are shown in (Table-II).

**Table II:** Distribution of CR and No CR in various clinicopathological groups.

Demographic Characteristics	No of Patients (N)	CR (n)	No CR (n)	P-value
**Gender**				
Male	34 (74%)	19 (55%)	15 (45%)	0.376
Female	12 (26%)	6 (50%)	6 (50%)	
Age				
Young (<40 years)	30 (65%)	20 (66%)	10 (34%)	0.048
Old (> 40 years)	16 (35%)	5 (31%)	11 (69%)	
**LDH**				
Low LDH (<400)	36 (78%)	22 (61%)	14 (39%)	0.018
High LDH (>400)	10 (22%)	3 (39%)	7 (61%)	
**TLC**				
High TLC (> 50,000)	18 (39%)	7 (39%)	11 (61%)	0.042
Low TLC (<50,000)	28 (61%)	18 (64%)	10 (36%)	
**ECOG Performance Status**				
Good (PS 0-1)	32 (69%)	20 (62%)	12 (38%)	0.008
Poor (PS 2)	14 (31%)	5 (35%)	9 (65%)	
**FAB Class**				
M1	6 (13%)	5 (83%)	1(17%)	
M2	12(26%)	7(58%)	5(42%)	
M4	9(19%)	4(44%)	5(56%)	
M5	3(6%)	1(34%)	2(66%)	
M6	4(8%)	1(25%)	3(75%)	
M7	2(4%)	1(50%)	1(50%)	
NOS	10(21%)	6(60%)	4(40%)	

## DISCUSSION

Acute myeloid leukemia (AML) is a heterogeneous hematological malignancy characterized by clonal expansion of myeloid blasts in the bone marrow and peripheral blood that leads to ineffective erythropoiesis and bone marrow failure.^9^ AML is most common among the adult population and accounts for approximately 80% of all cases. Despite advances in therapeutic regimens, the prognosis remains dismal in the elderly population.^10^ Induction chemotherapy is administered to improve the diseases baseline symptoms followed by CR.^11^ However, in some cases, consolidation chemotherapy is given to prevent the recurrence of the AML.^12^ Achievement of CR after chemotherapy usually comprises 3-4 cycles of cytarabine administered at high doses in combination with other chemotherapy medicines such as daunomycin, or idarubicin and etoposide to acquire high cure rate.^13^

In general, AML is more common in adults than in children. The incidence of AML increases with age. In the current study, it was found that 54% of male patients with <40 years of age were more responsive to induction therapy compared to non-responsive (46%) patients. The mean age of the study population was 35.02 years which was lower than the internationally reported age of 67 years.^14^,^15^ This may be due to the overall lower life expectancy in Pakistan which is only 67.2 years as compared to 78-80 years in developed countries.^16^ Another study supported nearly the same estimate of mean age where 55% were males and 45% females, with a mean age of 27.5+19.9 years.^10^ To be more specific, another study based on the FAB subtype showed the mean age for all AML (except AML-M4) to be between 27-29 years (for AML-M4, it was 45.60 years).^10^ The overall complete response rate of 54% is somewhat lower than reported clinical response rates of 50 to 75% in developed countries. There is no such study in Pakistan to assess clinical response after standard induction so far. However, a study was conducted in Sri Lanka to determine the demographic and other features of AML reveal CR of 50% in patients with AML.^17^ Additionally, a prospective study from India comparing the response rates in different groups after induction chemotherapy reported the CR or overall survival of 42.4% in elderly patients for a 2-year follow-up period.^18^ Our study also made almost similar observations and demonstrates that conventional chemotherapy approaches results are not being up to the standard for the great majority of patients with AML. Patients with a range of epithelial tumors may have elevated levels of LDH in their blood or neoplastic tissues. Studies demonstrate the elevated serum LDH level in patients with AML, compared to the health control at the time of admission. However, after four weeks the patients who responded to the chemotherapy showed decreased LDH value, compared to the non-responders who showed significant increases or no change in total LDH value.^19^,^20^ Almost similar observations were also made in our study that is patients with high LDH got bad outcomes (39% CR) as compared to those with low LDH (61%). Another interesting finding of our study is the relationship of high TLC level with poor AML prognosis and predicts severe outcomes in patients with AML which has been supported by various previous studies.^21^ Therefore, our study support that the development of more prognostic and predictive tools followed by advancement in therapeutic strategies and personalized medicine is essential to get a substantial response in patients with AML and to reduce disease burden.

### Limitations of our study:

First, it is not possible to quantify the degree to which selection bias (not getting high-risk patients of poor performance status or with comorbidities, extramedullary disease, CNS involvement) contributed to the observed improved outcome in the population of patients who proceeded to CR. Second, due to the cost involved, genetic factors cannot be commented upon, in addition to other factors. It was observed that older patients, for whom lower-intensity therapy was felt more appropriate, were at a higher risk of having refractory disease after two courses of induction chemotherapy. This underlines the importance of the development of either more effective, but well tolerated, chemotherapeutic agents, or improved delivery strategies such as the use of liposomal preparations. Third, the lower limit of the standard dose is given 60 mg daunorubicin (60-100mg) and100 mg cytarabine (100-200 mg). Despite these low doses, the achievement of similar outcomes (as in developed countries) is depicted in our population. It emphasizes the chemosensitivity of AML in the study. However, the data support the further exploration of sequential conditioning regimens which incorporate a cycle of intensive chemotherapy as an integral component of the preparative regimen for stem cell transplantation.

## CONCLUSION

The 3+7 Induction chemotherapy has 54% CR rates in patients with AML. In AML-M5, AML-M6, AML-NOS patients especially with high LDH and TLC and patients with advance age, CR rate is low and needs more aggressive treatment. Furthermore, advancement in AML therapy is needed to improve the survival rate.
